# Comparison of Smart Display Versus Laptop Platforms for an eHealth Intervention to Improve Functional Health for Older Adults With Multiple Chronic Conditions: Protocol for a Randomized Clinical Trial

**DOI:** 10.2196/64449

**Published:** 2025-04-03

**Authors:** David H Gustafson Sr, Marie-Louise Mares, Darcie C Johnston, John J Curtin, Klaren Pe-Romashko, Gina Landucci

**Affiliations:** 1 Center for Health Enhancement Systems Studies University of Wisconsin–Madison Madison, WI United States; 2 Department of Industrial and Systems Engineering University of Wisconsin–Madison Madison, WI United States; 3 Department of Communication Arts University of Wisconsin–Madison Madison, WI United States; 4 Department of Psychology University of Wisconsin–Madison Madison, WI United States

**Keywords:** eHealth, aged, geriatrics, functional health, multiple chronic conditions, smart display, smart speaker, primary care, quality of life

## Abstract

**Background:**

Maintaining functional health, or the ability to live independently, is a primary goal of individuals as they age, but most older adults develop chronic conditions that threaten this goal. Physical activity is a key aspect of self-care that can improve functional health, and digital interventions offering guidance on appropriate exercise can help. However, older adults with multiple morbidities may be unable to use a laptop or smartphone-based eHealth because poor vision, dexterity, mobility, or other physical challenges make typing or touch navigation difficult. A smart display platform—comprising a smart speaker plus a small visual screen—has the potential to remove these barriers because it is voice-activated.

**Objective:**

The study aims to compare usage patterns of an eHealth intervention for older adults when delivered via a voice-based smart display versus a typing-based laptop, and assess whether the smart display outperforms the laptop in improving functional health and its specific physical and mental aspects.

**Methods:**

A minimum of 356 adults aged 60 years and older with at least 5 chronic health conditions are to be recruited from primary care clinics and community organizations. Participants will be randomized 1:1 to 12 months of access to an evidence-based intervention, ElderTree, delivered on either a smart display or a touchscreen laptop, with a postintervention follow-up at 18 months. The primary outcome is differences between groups on a comprehensive measure of physical and mental functional health. Secondary outcomes are between-group differences in the subscales of functional health (eg, physical function and depression), as well as measures of health distress, loneliness, unscheduled health care, and falls. We will also examine mediators and moderators of the effects of ElderTree on both platforms. Participants will complete surveys at baseline, 6, 12, and 18 months, and ElderTree use data will be collected continuously during the intervention period in system logs. We will use linear mixed-effect models to evaluate outcomes over time, with treatment condition and time point as between-subjects factors. Separate analyses will be conducted for each outcome.

**Results:**

Recruitment began in July 2023 and was completed in May 2024, with 387 participants enrolled. The 12-month intervention period will end in May 2025; data collection will end in November 2025. Findings will be disseminated via peer-reviewed publications.

**Conclusions:**

Voice-activated digital health interventions have theoretical but untested advantages over typing-based technologies for older adults with physical limitations. As the population ages, and as multiple morbidities threaten the functional health of the majority of older adults, innovations in self-management are a matter of public health as well as individual quality of life.

**Trial Registration:**

ClinicalTrials.gov NCT05240534; https://clinicaltrials.gov/study/NCT05240534

**International Registered Report Identifier (IRRID):**

DERR1-10.2196/64449

## Introduction

### Background

A key measure of the physical and mental quality of life, functional health is the ability to perform basic personal care activities as well as more complex skills such as finances, transportation, and housekeeping: in essence, the ability to maintain one’s own agency [1]. Maintaining such agency, including living at home rather than in a care facility, is a primary goal of older adults [2]. Yet with age, most older adults develop chronic health conditions that erode their functional health and threaten their independence [3].

In the United States, chronic conditions such as diabetes, hypertension, and arthritis affect almost 95% of adults aged 60 years and older [4]. Among Medicare beneficiaries, about two-thirds have 3 or more chronic conditions and nearly one-fourth have 5 or more [5,6]. Treatment of co-occurring morbidities is complicated and costly given their complexity, prevalence, and ongoing nature [5,7]. For these same reasons, self-management is a vital aspect of patient care. Self-management includes skills and knowledge related to one’s health conditions, attention to emotional and cognitive states, and efforts related to lifestyle factors such as nutrition and exercise [8].

In its most recent Healthy People initiative, issued every 10 years since 1980 [9], the US Department of Health and Human Services (DHHS) declared a goal of improving the health and well-being among older adults. The first objective in meeting the goal is: “Increase the proportion of older adults with physical or cognitive health problems who get physical activity” [10]. According to health guidelines established by the Centers for Disease Control and Prevention [11], a consistent regimen of strengthening, balance, and aerobic activity can help older adults “feel, function, and sleep better; stay independent and fit so [they] can complete daily tasks; improve [their] mental health; and decrease pain and improve function”. Moreover, these beneficial impacts may be experienced immediately [12].

To accomplish this objective, labeled OA-01 (Older Adults-01), *Healthy People* *2030* recommendations include the use of digital health interventions that provide instruction and guidance in the form of “web-based interactive content” and “apps with goal-setting, activity tracking, and reminder functions” [13]. The initiative’s Community Preventive Services Task Force found that appropriately tailored home-based digital interventions with exercise content improved older adults’ balance, strength, and muscular endurance. In addition, people who used such interventions reported spending more time doing physical activity than they would have otherwise [14].

Digital health interventions have typically been delivered via computer websites (which require typing) or smartphone apps (which require touch navigation). Various academic and popular press accounts have suggested that smart speakers and smart displays, which also access the internet but are primarily navigated by voice rather than touch or typing, might be more accessible and appealing for older adults and others struggling with physical limitations [15-19]. Specifically, they have proposed that voice interactivity could alleviate challenges of hand tremors and vision loss and might seem more intuitive and companionable than a traditional app or website [20-22].

On the other hand, researchers have also suggested potential difficulties: age-related reductions in the volume, clarity, and pace of older adults’ speech may lead the device to misinterpret their questions and commands, and hearing loss may make it challenging for some older adults to understand the device’s responses [23-26]. More broadly, there is some indication that older adults may be less inclined to use technology-based interventions than to engage with traditional, in-person treatment. A 2023 meta-analysis of randomized trials of interventions for older adults’ functional mobility found slightly higher dropout rates for older adults assigned to technological interventions (eg, virtual reality exergaming, wearable devices, and telerehabilitation) than those assigned to conventional in-person rehabilitation or no treatment [27].

To summarize, it remains unclear whether voice interactivity versus touch or typing delivery of intervention will be more effective for older adults and help advance the objectives for older adults' health and physical activity outlined in *Healthy People 2030*. This paper reports on the protocol of a large clinical trial comparing the effectiveness of an eHealth intervention delivered on a smart display versus a laptop.

The intervention, ElderTree, is a web-based system that aims to improve the health and quality of life of older adults by offering interactive, informational, and motivational content and services such as health tracking, reminders, and social support. Developed by our Agency for Healthcare Research and Quality Center of Excellence in Active Aging, it was first tested in a randomized controlled trial (RCT) involving a community sample of 390 participants age 65+ years who received a laptop either with or without ElderTree access for 12 months [28]. In that study, participants in the ElderTree group who reported 3 or more primary care visits in the 6 months before baseline showed significantly better results on measures of mental quality of life, social support, and depression compared to control participants. Because primary care use is relatively high among patients who are managing multiple chronic conditions, these results suggest that patients with comorbidities may be most likely to benefit from a system such as ElderTree. A subsequent RCT testing a version of ElderTree among a clinic-based sample of older patients with at least 3 chronic conditions found socio-emotional health improvements, especially among women [29,30]. In the most recent RCT (ClinicalTrials.gov NCT04798196), ElderTree was adapted for older adults experiencing chronic pain and at least three comorbid conditions, with the primary aim of reducing pain interference. As in the current trial, participants were randomized to receive the intervention via a laptop or a smart display [31]. The outcome paper is currently in preparation.

The current study differs from the chronic pain study, by focusing on functional health among older adults with 5 or more chronic conditions. We recruited adults with an array of conditions known as metabolic syndrome (ie, vascular risk factors including hypertension, hyperglycemia, abdominal obesity, and dyslipidemia), for whom physical activity is of particular therapeutic import [32,33]. We increased the number of chronic conditions required to participate from 3 to 5. Almost a quarter (23%) of Medicare beneficiaries have 5 or more chronic conditions [34] and health risks and costs increase with the number of comorbid conditions [35,36]. As such, it is important to understand whether this sizable, high-risk, high-expenditure group is more likely to use and benefit from a voice-activated intervention for functional health, rather than a laptop version.

### Study Objectives

Although voice-activated systems seem to offer an easy, engaging way to use an intervention, it is an open question whether older adults coping with multiple chronic conditions would show more sustained use of a voice-activated platform and whether this would lead to improved health outcomes. To test this hypothesis, we propose a trial comparing the text and typing-based version of ElderTree delivered on a laptop to a voice-based version delivered on a smart display. The goal is to compare usage patterns of the two platforms and assess whether the smart display version outperforms the laptop in improving our primary outcome of functional health and associated secondary outcomes.

## Methods

### Trial Design

This is a nonblinded, 2-arm, parallel-design randomized trial, with participants allocated 1:1 to receive ElderTree on either a voice-based smart display or a text and touch-based laptop. The intervention period is 12 months, with surveys at baseline, 6, and 12 months, and a follow-up survey at 18 months.

### Sample Size and Study Setting

We planned for a minimum of 356 older adult participants to be recruited from both clinic and community settings throughout the state of Wisconsin.

### Study Arms

#### ElderTree Via Laptop

Participants in the ElderTree via Laptop (ET-LT) arm receive a touchscreen laptop computer, access to ElderTree, and Internet service for 12 months, and continue with their usual care. They will also receive US $10 for each completed survey, for a possible maximum of US $40.

#### ElderTree Via Smart Display

Participants in the ElderTree via Smart Display (ET-SD) arm continue with their usual care and receive a smart display, Internet service, and access to ElderTree for 12 months. The smart display consists of a voice-activated smart speaker and a small visual display that is optionally touch-activated. They will receive US $10 for each completed survey, for a possible maximum of US $40.

### Eligibility Criteria

Eligible participants must (1) be age 60 or older, (2) have at least 5 chronic conditions identified by the Centers for Medicare & Medicaid Services as prevalent among older adults [5,37], (3) among which 3 of which must be hypertension, hyperlipidemia, obesity, prediabetes, diabetes, or depression, (4) be willing to share medical record data about health care use (30-day hospital readmissions, emergency room urgent care, primary care, and specialty care visits), (5) allow researchers to share information with the patient’s primary care provider, (6) have no current psychotic disorder or form of dementia, (7) no acute medical problem requiring immediate hospitalization, (8) no need of an interpreter, and (9) no physical impairments preventing use of a computer or tablet.

### Recruitment

The Clinical and Health Informatics Institute at the University of Wisconsin–Madison (UW–Madison) searched clinic records from the Department of Family Medicine and General Internal Medicine system (UW Health) to identify patients meeting eligibility criteria and has sent eligible patient data (name, address, birth date, age, UW Health clinic location, primary care doctor) to the UW (University of Wisconsin) Office of Clinical Trials (OCT) via REDCap (Research Electronic Data Capture, Vanderbilt University). OCT then sent potential participants an opt-in letter describing the study, a consent form, and a stamped return letter inviting contact from the study team.

We supplemented recruitment from UW Health with additional clinic and community efforts aimed at achieving a diverse patient population. To increase our outreach to both participants of color and underserved patients, we worked with staff at Access Community Health Centers in Madison, who disseminated a recruitment flyer, consent form, and return card or opt-in to potentially eligible patients. In addition, advertisements on local television stations have been deployed in Madison, Milwaukee, Beloit, and other communities within driving distance, with specific outreach to older adults and African Americans. Last, through collaborations with leaders in the Black community, we reached out to churches, community centers, senior living communities, and other organizations, distributed a recruitment flyer and consent form with a return card or opt-in, placed advertisements in local publications, and conducted community sessions to introduce the study and invite participation.

Finally, we sent a one-time mass email to all current UW–Madison faculty and staff with information regarding the study, asking those interested to reply via email or call for more information. We also encouraged recipients to share the email with others they believed might be interested (eg, a parent or neighbor with multiple chronic conditions).

Regardless of recruitment method, when a return card or opt-in was received or a potential participant called, study staff assessed eligibility over the phone; provided a study overview that included potential benefits and risks, study procedures, and compensation; thoroughly walked through informed consent; conducted a brief exercise screen to allow tailoring of exercise content [38]; and addressed questions. If the individual met eligibility criteria, the baseline survey was mailed; community participants also received the consent form, while clinic participants had already received it with their opt-in letter. Patients were given as much time as needed to decide whether to participate and had the option for a follow-up call after reviewing the materials. The baseline survey takes 20-30 minutes to complete. Measures are the same in both arms to avoid differential dropout. As the trial progresses, we document those who choose not to participate and why, following the CONSORT (Consolidated Standards of Reporting Trials). Patients can opt to stop their participation at any time by contacting the research team.

### Randomization

The project manager used a computer-generated allocation sequence to randomize participants on a 1:1 ratio to ET-SD or ET-LT, stratified on gender (male or female), number of chronic conditions (5-7 or 8+), and recruitment site. There are 7 site variables: 3 UW Health clinics, the Access Community Health clinic site, and 3 communities (Madison, Milwaukee, and Beloit). Once an individual verbally consented, a home visit was scheduled to collect the baseline survey, randomize, determine the preferred location of the laptop or smart display, and set up the device. Staff then conducted training on the assigned intervention. If no visit was desired, equipment could be shipped after the completed baseline survey was mailed and received, and technology setup and training could be conducted by phone. The research team is available during usual operating hours for participants to contact them with questions or issues.

Once an assignment has been made, it is of course not possible to blind participants to their condition. Further, in order to set up participants on their assigned system, the researcher conducting the training also could not be blind to the condition after the assignment.

### Timeline

Table 1 shows the timeline by year of the study, with year 1 beginning in August 2021 and year 5 ending in July 2026.

**Table 1:** Timeline of key project activities.

Activity	Timeline
Develop and test ElderTree intervention on the smart display	Year 1, months 2-11
Develop content plan for ElderTree on both platforms	Year 1, months 5-9
Prepare and finalize study and data collection materials	Year 1, months 7-9
Obtain institutional review board approvals	Year 1, month 8
Finalize data quality monitor plan	Year 1, month 9
Train research staff	Year 1, months 9-10
Recruit and randomize patients	Year 1, month 9 to year 3, month 10
Produce and manage ElderTree content for both systems	Year 1, month 9 to year 5, month 4
Collect data	Year 1, month 9 to year 5, month 4
Clean and prepare data	Year 2, month 4 to year 5, month 6
Analyze results	Year 5, months 7-10
Publish	Year 5, months 11-12

### Intervention

#### System Overview

ElderTree is one of a suite of eHealth systems developed by our Center and collectively known as CHESS (Comprehensive Health Enhancement Support System). Like other CHESS systems, ElderTree is built on principles of continuing care and self-management, such as long duration [39], tracking [40], prompts [41], goal setting and action planning [42], problem-solving and self-tailoring [43], and social support [44]. In randomized trials, CHESS systems have significantly improved risky drinking [45]; asthma control [46]; quality of life and cost of care in human immunodeficiency virus patients [47]; quality of life and self-efficacy in breast cancer patients [48-50]; and caregiver burden, symptom distress, and median length of survival in patients with lung cancer [51]. All CHESS systems, including ElderTree, are consistent with self-determination Theory, which asserts that satisfying these 3 basic psychological needs contributes to adaptive functioning: competence (feeling effective or not overwhelmed), social relatedness (feeling connected to others or not isolated), and intrinsic motivation (feeling autonomous about behavioral changes or not coerced) [52].

ElderTree is a multifaceted system focused on older adults’ physical and mental health and quality of life. As reported in earlier ElderTree studies [28-30], the laptop system is a members-only website free of ads, with design features based on older users’ feedback and best practices for older populations (eg, larger fonts or uncluttered screens) to optimize comprehension, navigation, and usability [53]. The ET-SD version follows these usability principles as well. Both systems substantially replicate ElderTree as described in an earlier study comparing ET-LT to an attention control [29,30], with modifications and enhancements to focus on the functional health of individuals with multiple morbidities and to align with the technical capabilities of the smart display platform.

#### System Content

ElderTree offers video content in the areas of functional movement, information and advice, mood and mental health, and entertainment, as well as social engagement and health tracking features. Video content is both originally produced and curated from high-quality external sources. Descriptions of each feature or function are listed in Table 2, and the home screen organization for both platforms is shown in Figure S1 in Multimedia Appendix 1: ElderTree screens and physical activity decision tree.

**Table 2:** Key features of the ElderTree eHealth intervention.

Feature or function	Description
“Let’s Move” videos	Functional movement videos, in 3 assigned ability groupings based on participants’ physical health screening and doctor approval.
“Tips & Info” videos	Short, practical information and advice on a wide range of topics related to physical and mental health (eg, “How Blood Pressure Works”, “Finding Friends as an Older Adult”, “Easy Ideas for Healthy Eating”).
“Relax & Inspire” videos	Meditation, relaxation, and breath work videos for sleep, reducing stress, creativity, and more.
“Just for Fun” videos	Humor, music, and human interest are for pleasure (this content is located in “Relax & Inspire” on the smart display platform).
CalmConnect multisensory videos [54]	Program of short practice videos that combine rhythm, hand movement patterns, and facial expression cues, engaging the user physically, cognitively, and emotionally with the goal of strengthening social connectivity and mental health.
Wednesday Meetup	One-hour weekly facilitated group video meeting with a topic presentation (eg, balance, falls, and vision health) and small breakout rooms for socializing.
Weekly Survey	Tracking of 10 self-reported general health indicators (eg, sleep, mood, exercise), with results presented in graph form over time.
Thought of the Day	A new motivational or inspirational quote delivered daily at with the user’s first visit to the intervention.
Prompts	Reminders to take the weekly health tracking survey; notifications of new content.
Featured content	On the home screen, new and existing content is highlighted to encourage exploring services and resources, and is continually rotated to keep ElderTree fresh.
Favorites	Bookmarking and retrieval feature.
System tutorials	Instructional videos for accessing ElderTree features and content.

The critical physical exercise content is located in ElderTree’s “Let’s Move” area, offering videos targeting balance, strength, endurance, and flexibility. After randomization, participants in both conditions were placed in one of three activity-level groups on the basis of their responses to the Exercise and Screening for You (EASY) Tool [38] and approval from their primary care physician (PCP), to ensure safety and accessibility. Figure S2 in Multimedia Appendix 1 shows the decision tree for assigning each participant to their group within the study arm. The three groups are as follows:

1. Group 0: restricted based on the EASY Tool and do not have approval from their PCP to use standing activity content on ElderTree. The fitness activities available to them are limited to gentle seated movements for coordination, dexterity, and mental stimulation (eg, “Wake Up Your Brain”). Also, in Let’s Move, Group 0 has access to an 8-module course on managing chronic pain, developed by our research team.

2. Group 1: restricted based on the EASY Tool, but their PCP has given approval to engage in supported standing activities. For this group, we developed a collection of instructional and practice videos with a geriatrician expert in functional movement for older adults who have health conditions. The videos are available in all areas of fitness: balance, flexibility, strength, and endurance. Group 1 participants must view a safety video for each of the 4 fitness categories before access to the movement videos is unlocked. Group 1 also has access to all Group 0 content.

3. Group 2: no restrictions, based on the EASY Tool, and may use all the movement videos on ElderTree after viewing the safety videos in each of the 4 fitness categories. The Group 2 content includes all activities available to Groups 0 and 1, as well as videos provided by outside experts in fitness and functional movement for older adults. These externally sourced videos have been vetted by the study’s geriatrician for safety, suitability, and quality. Playback times and challenge levels are indicated in every video title to enable users to self-tailor their selections on any given day.

### Outcomes

**Figure 1:**
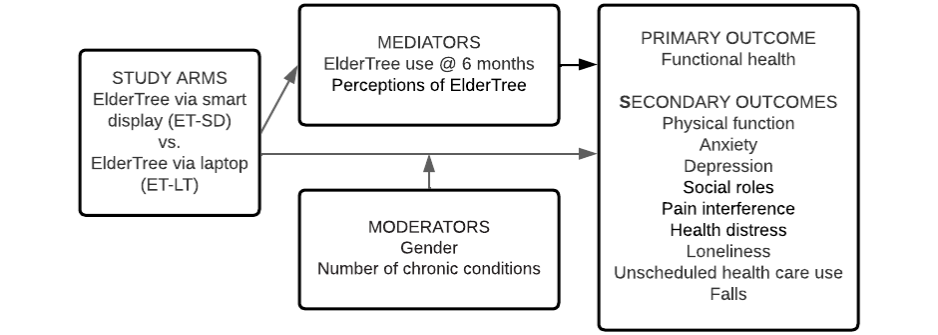
Study logic.

The primary outcome is differences between groups over time in functional health, a multifaceted variable that includes physical function, pain interference and intensity, fatigue, sleep disturbance, anxiety, depression, and satisfaction with one’s social roles and activities. To tease apart these elements, the secondary outcomes include the functional health subscales of physical function, pain interference, anxiety, depression, and social role satisfaction. Additional secondary outcomes are health distress, loneliness, unscheduled health care use, and falls. Figure 1 diagrams the study logic.

### Measures

#### Primary Outcome

The 29-item PROMIS (Patient-Reported Outcomes Measurement Information System)-29 v2.1 is used to measure functional health [55]. Items tapping physical function, pain interference with aspects of daily life, anxiety, depression, ability to participate in social roles and activities, fatigue, and sleep disturbance are scored on 5-point Likert scales, while a single 11-point item (range=0–10) rates pain intensity. The possible ranges, scored by providers of the measure [56], are 21.2–67.6 for a social-emotional cluster and 16.2–67.7 for a physical cluster of functional health, with higher scores indicating better functioning.

#### Secondary Outcomes

We are using PROMIS-43 v2.1 subscales to separately assess physical function, pain interference, anxiety, depression, and satisfaction with social roles and activities [57]. For each subscale, participants respond on a 5-point (1-5) Likert scale to 6 items modified from occurring “currently” to “in the past 7 days,” for consistency with other survey items. For the physical function items (eg, “Are you able to go for a walk of at least 15 minutes?”), higher scores indicate poorer function (total possible range=22.9-56.9). For pain interference (eg, “How much did pain interfere with the things you usually do for fun?”), higher scores indicate greater interference (total possible range=41.6-75.6). Anxiety items include “I felt nervous” and “My worries overwhelmed me”; higher scores indicate greater anxiety (total possible range=40.3-81.6). Depression items include, “I felt like a failure” and “I felt unhappy”; higher scores indicate more depression (total possible range=41-79.4). For social roles items (eg, “I have to limit my regular activities with friends”), higher scores indicate more difficulty or limitation (total possible range=29-64.1).

Health distress is measured with the 4-item Lorig Health Distress Scale [58,59]. Sample items are how often in the past month a participant has been “discouraged by your health problems” and “fearful about your future health.” Higher scores on the 6-point (0–5) Likert scale indicate greater distress. The total possible score is the mean of the 4 items (range=0 5).

Loneliness is assessed with the 5 Loneliness scale items in the NIH Toolbox Social Relationship scales item bank v2.0 [60]. Sample items include how often in the past month a participant has felt “alone and apart from others” and “left out”. Scores are calculated by the scale developer; the possible range=37–85, with higher scores indicating greater loneliness.

For unscheduled health care visits, participants will report at each survey time point their number of urgent care clinic visits, emergency room visits, and overnight hospital stays in the past month.

The number of falls in the past 6 months, as well as the number requiring medical attention, will also be surveyed at each time point. A fall is defined in the survey as “when your body goes to the ground without being pushed.”

Measures for all planned outcomes and their psychometrics are presented in Table 3.

**Table 3:** Outcome measures, the number of survey items, and psychometrics.

Variable		Measure	Items, n	Psychometrics
**Primary outcome**				
	Functional health	PROMIS^a^-29 v2.1	29	α≥.94 [55]
**Secondary outcomes**				
	Physical function	PROMIS-43 physical function subscale	6	α>.9 [57]
	Pain interference	PROMIS-43 pain interference subscale	6	α>.9 [57]
	Anxiety	PROMIS-43 anxiety subscale	6	α>.9 [57]
	Depression	PROMIS-43 depression subscale	6	α>.9 [57]
	Social roles	PROMIS-43 social roles subscale	6	α>.9 [57]
	Health distress	Lorig Health Distress Scale	4	α>.87 [58]
	Loneliness	NIH Toolbox	5	α≥.93 [60]
	Number of unscheduled health care visits	N/A^b^	3	N/A
	Falls	N/A	2	N/A

^a^PROMIS: Patient-Reported Outcomes Measurement Information System.

^b^N/A: not applicable.

#### Mediation, Moderation, and Covariates

We will test whether functional health at 12 months is mediated by participants’ amount of ElderTree use and perceptions of ElderTree at 6 months. ElderTree use will be assessed via continuously collected system data. For perceptions of ElderTree, participants rate 18 items (eg, “It is easy to use,” “It helped me learn more about my health issues”) on a 4-point (1-4) Likert scale and answer 6 open-ended questions (eg, “What stopped you or hindered you from using ElderTree or the computer or smart system in general?”). We will also examine possible moderation due to gender and the number of chronic conditions at baseline. Finally, as potential covariates we will test baseline race and ethnicity, education level, and barriers to technology due to physical limitations (eg, vision or hearing challenges); those that significantly predict our primary outcome will be included in the final analysis.

### Power Analysis and Sample Size

The primary hypothesis is that the intervention modality (voice activation vs typing and touching) will affect functional health. Based on PROMIS validation studies (eg, chronic pain, arthritis, and cancer), a difference of 3 points or more between study arms is considered to indicate a clinically meaningful difference [61]. The PROMIS website also gives the mean and SD of the T-score metric (mean 50, SD 10). Power calculations for this primary hypothesis were performed using the SAS procedure PROC GLMPOWER (SAS Version 9.4) [62]. Results indicated that 282 participants after attrition would be needed for 80% power (*P*<.05) to observe a difference score of 3 points between the two study arms at 12 months. In our original ElderTree study, 79% of the sample completed the 12-month survey [28], and in the subsequent study, 92% of participants did so [30]. We based our sample size on the more conservative numbers from the original study and planned to recruit a total of 356 participants, or 178 per arm, to achieve 282 after attrition.

With regard to power for moderation analyses, we ran a sensitivity analysis to compute the smallest effect size our study would be powered to find using 280 given 4 groups (eg, 2 study arms × 2 genders). With N=280, G*Power calculations [63] show our study would have a 95% chance (with *P*<.05) of observing a moderation effect as small as a Cohen *d* of 0.22.

### Data Collection

#### Patient Surveys

Patients will complete surveys at baseline, 6, 12, and 18 months. Baseline surveys were collected at training and setup, and subsequent surveys will be mailed to patients with a return stamped envelope. Participants are encouraged to contact the study staff if questions arise. All 4 surveys will assess primary and secondary outcomes using the measures detailed above. At 6, 12, and 18 months, participants will also respond to the checklist of chronic conditions used for eligibility screening as well as answer items assessing their perceptions of the intervention. Demographics were gathered only at baseline.

#### ElderTree System Data

ElderTree usage data are automatically and continuously collected in time-stamped log files, including logins, services used, pages accessed, and weekly surveys completed.

In addition, data are collected from the weekly survey of self-rankings for medication adherence, falls, thinking and memory, mood, healthy meals, snacks and drinks, physical activity, quality time with others, sleep, pain, and balance.

### Retention

We are promoting study retention by providing telephone and email support for patients’ use of the technologies and by following up with patients to encourage them to return surveys. If a survey is not returned within 2 weeks, a research team member will call to check that the survey was received and encourage the patient to complete and return it in the provided envelope. The date and time of the phone call will be recorded in REDCap, along with whether the researcher talked to the participant directly (vs left a message) and any information gathered during the conversation. If we cannot reach the participant, another copy of the survey will be sent with a personal note asking them to complete it or call us on our toll-free number if they have questions or are no longer interested.

### Data Quality and Management

Surveys comprise standardized measures that have been validated for older adults. In addition, the following strategies, developed during our previous large RCTs with this population, are implemented to reduce errors and missing data: (1) surveys are sent via hard copy, with paid return envelopes; (2) visual formatting is simple and clear, in large print on uncluttered pages; (3) tables of items and response options are designed with line-by-line alternate shading for easy tracking; and (4) instructions are explicit as to how participants should respond if they feel a question does not apply or they do not know the answer. Participants are encouraged to call in with questions. Once a survey is returned, trained research staff will evaluate it within a week of receipt; if data issues are found (eg, missing responses, multiple responses to a single item), staff will contact the participant to resolve the error.

To mitigate the risk of breaches in patient confidentiality, all participants are assigned a unique code number. Participant contact information and survey data are housed electronically in REDCap. Survey data are double-entered by two different individuals to ensure accuracy. Paper files are stored in a locked room in locked cabinets and can be accessed only by authorized personnel. The database administrator provides access to study data at appropriate levels for various members of the research team.

### Statistical Methods

#### Predictor and Outcome Assumptions

Participants were assigned at random, with constraints that study arms would have roughly equal proportions for key covariates (eg, race, education, baseline number of chronic conditions). The effect of key covariates on outcomes will be assessed for each model. We will assess whether there are main effects of recruitment sites or interactions with the study arm (ie, whether data can be pooled across sites). Variables will be examined using standard summary statistics, visualizations, and tests for normality and homoscedasticity. Data will be transformed as needed.

#### Missing Data

With regard to survey data, in the previous ElderTree RCT of older adults coping with at least 3 high-risk chronic conditions, 320 of 344 participants (93%) completed the 6-month survey, 309 (89.8%) completed the 12-month survey, and 298 (86.6%) participants completed the 18-month survey [30]. In completed surveys, data were missing on about 2% of core items. Occasional out-of-residency time is also expected for participants (eg, travel and hospitalizations). We encourage patients to take the intervention with them when possible, but absences without ElderTree will be addressed as covariates in our outcome models.

We expect rates of missing data in this study to be similar to those reported above for the ElderTree RCT. We will report rates of missing data by study arm and time point. We will also use logistic regression to predict missing data as part of our evaluation of missing data patterns.

#### Effects of Study Arm on Outcomes

Primary and secondary outcomes will be analyzed using an intention-to-treat approach that includes all observed data from all participants who were randomized to a study arm. These analyses will follow a model-based approach that assumes missing data for outcomes at specific time points are missing at random (MAR) [64]. Specifically, a linear mixed model [65-68] will be used to examine the effects of the study arm (ET-SD vs ET-LT, a between-subjects factor) on the primary outcome, functional health, over time. Linear mixed models will also assess the effects of the study arm on each of the secondary outcomes over time (ie, an interaction of the between-subjects factor of the study arm and the within-subjects factor of time of measurement). Count variables that lack sufficient variability to be treated as count data (negative binomial distribution) will be treated as dichotomous variables and run using a binomial distribution in the linear mixed model.

Supplemental sensitivity analyses [69] will also be conducted to assess the robustness of conclusions to our assumption that missing data are MAR. Specifically, we will use comparable linear mixed-effects models to examine the effects of the study arm using only complete cases, as would be appropriate if data were missing completely at random. To address the possibility that the data are missing not at random, we will use both the Diggle-Kenward approach (which assumes that dropout is a function of time-specific outcomes) and the Wu-Carroll approach (which assumes that dropout is a function of one’s developmental trajectory) [66-68,70].

#### Mediation of Effects of Study Arm on Primary Outcome

We predict effects of the study arm on functional health will be mediated by the amount of ElderTree use and perceptions of ElderTree at the 6-month midpoint. We will use structural equation modeling with separate models for the social-emotional and physical components of functional health.

#### Moderation of Study Arm on Primary Outcome

We predict that the effects of the study arm on functional health will be moderated by gender and the number of chronic conditions at baseline. We will use linear mixed models, testing each moderator separately, to examine the moderator-by-arm interaction while accounting for time.

### Ethical Considerations

This study protocol received ethical approval from the University of Wisconsin Health Sciences and Minimal Risk Research institutional review board (reference number 2020-0984) and has been registered at ClinicalTrials.gov (NCT05240534). Consent forms were mailed to potential participants and research staff walked through informed consent over the phone. Participation was voluntary and participants could opt out at any time. Only a small number of research staff has access to patient data and all outcomes and usage data are deidentified. Participants were paid US $10 for each outcome survey completed and received a free laptop or smart display and internet service for 12 months if needed.

## Results

**Figure 2:**
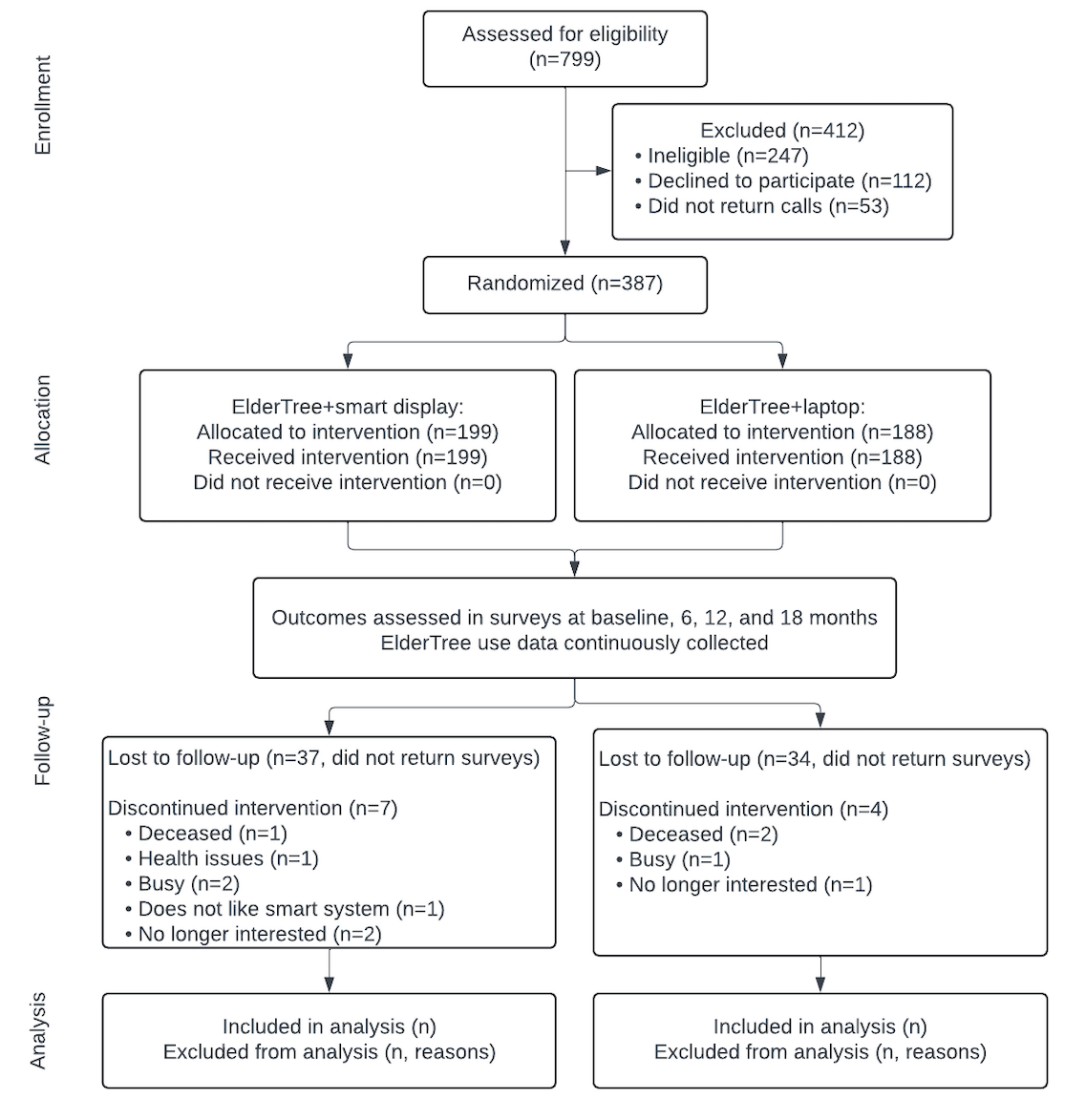
CONSORT (Consolidated Standards of Reporting Trials) flow diagram.

As of May 2024, a total of 387 new participants have been enrolled, concluding recruitment 2 months earlier than originally scheduled. The 12-month intervention period will end in May 2025, and data collection will end in November 2025. Findings will be disseminated via peer-reviewed publications. Figure 2 shows the status of participants throughout the trial. Interim numbers are shown in the Follow-up section, as the intervention and data collection are not yet complete. The final numbers for the Analysis section will be reported in the main outcome publication.

## Discussion

### Anticipated Findings

The central question of this study is whether a voice-activated smart system will be better than a laptop for delivering an intervention for functional health to older adults with ≥5 chronic conditions. The prediction is that those randomized to access the intervention via a voice-activated smart display will show more use of the intervention than those randomized to access it via a laptop and that greater use will lead to more positive change over time in the primary and secondary health-related outcomes.

Few studies thus far have rigorously tested smart displays as a platform for health interventions for older adults. A 2024 scoping review found 22 studies (published between 2010 and 2022) examining community-dwelling older adults’ use of personal voice assistants [26]. The overall conclusion was that many older adults found the devices convenient and easier to use than a computer for basic tasks like reminders or checking the weather, but they sometimes struggled to make themselves understood or to remember the necessary commands. As the authors noted, all of these studies were exploratory, and none examined effects on quality of life or health-related outcomes.

Since then, 2 studies have examined socio-emotional outcomes of older adults’ uses of smart devices, but one lacked any comparison group (examining pre-post changes only) [71], and the other compared a smart speaker (audio only) versus a smart display (audio + screen) [72]. While both found that frequent use of smart systems was associated with positive socio-emotional changes (eg, reductions in loneliness), it is possible the same changes would have occurred with laptop use. A third, nonrandomized study examined whether a smartphone health intervention for older adults would be more effective if participants agreed to add a smart speaker with extra functions (eg, medication reminders, exercise programs, quizzes) [73]. There were no differences between the 2 self-selected groups in changes over time in depression or 5 of 6 health outcomes; only dietary diversity showed greater improvements in the group that agreed to have the smart speaker as well as the smartphone.

In sum, despite claims about the possibility that voice interactivity may increase the accessibility and effectiveness of health interventions for older adults, our project is one of the first randomized trials comparing a smart display versus a laptop for delivering an intervention for older adults, who face complex health challenges. As in any study, there are strengths and limitations.

The first strength lies in the methodological rigor involved in a randomized trial that followed 356 older adults over 12 months with a postintervention follow-up at 18 months. Another strength is that we can gather objective, ongoing data about participants’ uses of the intervention, rather than relying on their recollections. Next is our recruitment of older adults with 5 or more comorbid conditions, given the need for effective interventions that will engage this sizable, high-risk population. In addition, the focus on functional health is a core determinant of older adults’ ability to remain independent.

There are also a number of limitations. One is that this iteration of ElderTree, like those that came before it, was developed before the widespread availability of large language models and artificial intelligence (AI) interactivity. Although participants can interact with ElderTree on the smart speaker by voice, they must do so using specific commands, rather than the intuitive, conversational exchanges now made possible by AI. A second limitation is the absence of a randomized control group that does not receive access to ElderTree: we prioritized adequate power to detect a difference between the 2 intervention groups but will not be able to assess whether both improve outcomes relative to treatment as usual. Next is that we rely on participants’ perceptions of their functional health, rather than using clinical data or direct assessment of their functional movement. We regard these limitations as invitations for further research, particularly research that would probe older adults’ uses and responses to digital devices that are much more interactive and responsive to voice commands, given AI advances.

### Conclusion

Whether the theoretical advantages of voice activation translate to improved effectiveness of eHealth interventions has yet to be determined. Although technologies are constantly developing, the issue of talking versus typing for health interventions for older adults is fundamental and supersedes specific devices. In this way, if ElderTree on the smart display does outperform the laptop in terms of use and health-related outcomes, this would have important implications for the design of future health interventions for this population.

## Appendix

Multimedia Appendix 1 ElderTree screens and physical activity decision tree.

Multimedia Appendix 2 SPIRIT (Standard Protocol Items: Recommendations for Interventional Trials) checklist.

## Abbreviations

AI: artificial intelligence
CHESS: Comprehensive Health Enhancement Support System
CONSORT: Consolidated Standards of Reporting Trials
DHHS: Department of Health and Human Services
EASY: Exercise and Screening for You
ET-LT: ElderTree on a laptop platform
ET-SD: ElderTree on a smart display platform
MAR: missing at random
OA-01: Older Adults-01
OCT: Office of Clinical Trials
PCP: primary care physician
PROMIS: Patient-Reported Outcomes Measurement Information System
RCT: randomized controlled trial
REDCap: Research Electronic Data Capture
UW Health: University of Wisconsin–Madison Department of Family Medicine and General Internal Medicine system
UW: University of Wisconsin
UW–Madison: University of Wisconsin—Madison

## Data Availability

Following the completion of the final analysis, the datasets generated or analyzed during this study will be available from the corresponding author on reasonable request.

## References

[1] Edemekong PF, Bomgaars DL, Sukumaran S, Schoo C (2023). Activities of Daily Living.

[2] Molzahn A, Skevington SM, Kalfoss M, Makaroff KS (2010). The importance of facets of quality of life to older adults: an international investigation. Qual Life Res.

[3] Rejeski WJ, Brawley LR (2006). Functional health: innovations in research on physical activity with older adults. Med Sci Sports Exerc.

[4] (2024). The top 10 most common chronic conditions in older adults. National Council on Aging.

[5] (2012). Chronic conditions among medicare beneficiaries: Chartboook 2012 edition. Centers for Medicare & Medicaid Services.

[6] Wolff JL, Starfield B, Anderson G (2002). Prevalence, expenditures, and complications of multiple chronic conditions in the elderly. Arch Intern Med.

[7] Lehnert T, Heider D, Leicht H, Heinrich S, Corrieri S, Luppa M, Riedel-Heller S, König HH (2011). Review: health care utilization and costs of elderly persons with multiple chronic conditions. Med Care Res Rev.

[8] Lorig KR, Sobel DS, Ritter PL, Laurent D, Hobbs M (2001). Effect of a self-management program on patients with chronic disease. Eff Clin Pract.

[9] (2023). Healthy people 2030: questions and answers. Office of Disease Prevention and Health Promotion.

[10] (2021). Healthy people 2030: older adults: overview and objectives. Office of Disease Prevention and Health Promotion.

[11] (2023). Older adult activity: an overview. Physical Activity Basics.

[12] (2024). Living with a chronic condition. Chronic Disease.

[13] (2019). Physical activity: digital health interventions for adults 55 years and older. Office of Disease Prevention and Health Promotion.

[14] (2022). Physical activity: home-based exercise interventions for adults aged 65 years and older. Community Guide.

[15] Capan F (2016). Why amazon device is a gift for healthcare. Viewpoint.

[16] (2017). Amazon echo voice commands offer big benefits to users with disabilities. Consumer Reports.

[17] Thompson AC (2017). Persuading your older parents to take the smart home leap. CNET.

[18] Woyke E (2017). The octogenarians who love Amazon's Alexa. MIT Technology Review.

[19] (2018). Amazon echo for dementia: technology for seniors. DailyCaring.

[20] McCloud R, Perez C, Bekalu MA, Viswanath K (2022). Using smart speaker technology for health and well-being in an older adult population: pre-post feasibility study. JMIR Aging.

[21] Edwards KJ, Jones RB, Shenton D, Page T, Maramba I, Warren A, Fraser F, Križaj T, Coombe T, Cowls H, Chatterjee A (2021). The use of smart speakers in care home residents: implementation study. J Med Internet Res.

[22] Sin J, Munteanu C, Ramanand N, Rong Tan Y (2021). VUI influencers: How the media portrays voice user interfaces for older adults. https://doi.org/10.1145/3469595.3469603.

[23] Werner L, Huang G, Pitts BJ (2019). Automated speech recognition systems and older adults: a literature review and synthesis. Proc Hum Factors Ergon Soc Annu Meet.

[24] Werner L, Huang G, Pitts B (2022). Smart speech systems: a focus group study on older adult user and non-user perceptions of speech interfaces. Int J Hum-Comput Interact.

[25] Pradhan A, Lazar A, Findlater L (2020). Use of intelligent voice assistants by older adults with low technology use. ACM Trans Comput-Hum Interact.

[26] Arnold A, Kolody S, Comeau A, Miguel Cruz A (2024). What does the literature say about the use of personal voice assistants in older adults? A scoping review. Disabil Rehabil Assist Technol.

[27] Cieślik B, Mazurek J, Wrzeciono A, Maistrello L, Szczepańska-Gieracha J, Conte P, Kiper P (2023). Examining technology-assisted rehabilitation for older adults' functional mobility: a network meta-analysis on efficacy and acceptability. NPJ Digit Med.

[28] Gustafson DH, Kornfield R, Mares M, Johnston DC, Cody OJ, Yang EF, Gustafson DH, Hwang J, Mahoney JE, Curtin JJ, Tahk A, Shah DV (2022). Effect of an eHealth intervention on older adults' quality of life and health-related outcomes: a randomized clinical trial. J Gen Intern Med.

[29] Gustafson DH, Mares M, Johnston DC, Mahoney JE, Brown RT, Landucci G, Pe-Romashko K, Cody OJ, Gustafson DH, Shah DV (2021). A web-based eHealth intervention to improve the quality of life of older adults with multiple chronic conditions: protocol for a randomized controlled trial. JMIR Res Protoc.

[30] Gustafson DS, Mares M, Johnston D, Vjorn O, Curtin J, Landucci G, Pe-Romashko K, Gustafson DH (2024). An eHealth intervention to improve quality of life, socio-emotional, and health-related measures for older adults with multiple chronic conditions: a randomized controlled trial. JMIR Preprints.

[31] Gustafson DH, Mares M, Johnston DC, Landucci G, Pe-Romashko K, Vjorn OJ, Hu Y, Gustafson DH, Maus A, Mahoney JE, Mutlu B (2022). Using smart displays to implement an eHealth system for older adults with multiple chronic conditions: protocol for a randomized controlled trial. JMIR Res Protoc.

[32] Silveira Rossi JL, Barbalho SM, Reverete de Araujo R, Bechara MD, Sloan KP, Sloan LA (2022). Metabolic syndrome and cardiovascular diseases: going beyond traditional risk factors. Diabetes Metab Res Rev.

[33] Alizaei Yousefabadi H, Niyazi A, Alaee S, Fathi M, Mohammad Rahimi GR (2021). Anti-inflammatory effects of exercise on metabolic syndrome patients: a systematic review and meta-analysis. Biol Res Nurs.

[34] Pena MT, Mohamed M, Burns A, Fuglesten BJ, Ochieng N, Chidambaram P (2023). A profile of medicare-medicaid enrollees (dual eligibles). KFF.

[35] Cavalli L, Angehrn L, Schindler C, Orsini N, Grob C, Kaufmann M, Steiner LA, Schwenkglenks M, Dell-Kuster S (2022). Number of comorbidities and their impact on perioperative outcome and costs - a single centre cohort study. Swiss Med Wkly.

[36] Kuwabara K, Imanaka Y, Matsuda S, Fushimi K, Hashimoto H, Ishikawa KB, Horiguchi H, Hayashida K, Fujimori K (2008). The association of the number of comorbidities and complications with length of stay, hospital mortality and LOS high outlier, based on administrative data. Environ Health Prev Med.

[37] (2023). Data snapshots. Centers for Medicare & Medicaid Services.

[38] (2021). Exercise and screening for you: EASY tool. Easy For You.

[39] Maisto SA, Zywiak WH, Connors GJ (2006). Course of functioning 1 year following admission for treatment of alcohol use disorders. Addict Behav.

[40] Scherr D, Kastner P, Kollmann A, Hallas A, Auer J, Krappinger H, Schuchlenz H, Stark G, Grander W, Jakl G, Schreier G, Fruhwald FM, MOBITEL Investigators (2009). Effect of home-based telemonitoring using mobile phone technology on the outcome of heart failure patients after an episode of acute decompensation: randomized controlled trial. J Med Internet Res.

[41] Davis JR, Glaros AG (1986). Relapse prevention and smoking cessation. Addict Behav.

[42] van Osch L, Lechner L, Reubsaet A, Wigger S, de Vries H (2008). Relapse prevention in a national smoking cessation contest: effects of coping planning. Br J Health Psychol.

[43] Lorig KR, Holman H (2003). Self-management education: history, definition, outcomes, and mechanisms. Ann Behav Med.

[44] Shaw BR, Yeob Han J, Hawkins RP, McTavish FM, Gustafson DH (2008). Communicating about self and others within an online support group for women with breast cancer and subsequent outcomes. J Health Psychol.

[45] Gustafson DH, McTavish FM, Chih M, Atwood AK, Johnson RA, Boyle MG, Levy MS, Driscoll H, Chisholm SM, Dillenburg L, Isham A, Shah D (2014). A smartphone application to support recovery from alcoholism: a randomized clinical trial. JAMA Psychiatry.

[46] Gustafson D, Wise M, Bhattacharya A, Pulvermacher A, Shanovich K, Phillips B, Lehman E, Chinchilli V, Hawkins R, Kim J (2012). The effects of combining web-based eHealth with telephone nurse case management for pediatric asthma control: a randomized controlled trial. J Med Internet Res.

[47] Gustafson DH, Hawkins R, Boberg E, Pingree S, Serlin RE, Graziano F, Chan CL (1999). Impact of a patient-centered, computer-based health information/support system. Am J Prev Med.

[48] Gustafson DH, McTavish F, Hawkins R, Pingree S, Arora N, Mendenhall J, Simmons GE (1998). Computer support for elderly women with breast cancer. JAMA.

[49] Gustafson DH, Hawkins R, Pingree S, McTavish F, Arora NK, Mendenhall J, Cella DF, Serlin RC, Apantaku FM, Stewart J, Salner A (2001). Effect of computer support on younger women with breast cancer. J Gen Intern Med.

[50] Gustafson DH, Hawkins R, McTavish F, Pingree S, Chen WC, Volrathongchai K, Stengle W, Stewart JA, Serlin RC (2008). Internet-based interactive support for cancer patients: are integrated systems better?. J Commun.

[51] Gustafson DH, DuBenske LL, Namkoong K, Hawkins R, Chih M, Atwood AK, Johnson R, Bhattacharya A, Carmack CL, Traynor AM, Campbell TC, Buss MK, Govindan R, Schiller JH, Cleary JF (2013). An eHealth system supporting palliative care for patients with non-small cell lung cancer: a randomized trial. Cancer.

[52] Ryan RM, Deci EL (2000). Self-determination theory and the facilitation of intrinsic motivation, social development, and well-being. Am Psychol.

[53] Gustafson DH, Maus A, Judkins J, Dinauer S, Isham A, Johnson R, Landucci G, Atwood AK (2016). Using the NIATx model to implement user-centered design of technology for older adults. JMIR Hum Factors.

[54] (2024). What is calmconnect?. PrioHealth.

[55] Hays RD, Spritzer KL, Schalet BD, Cella D (2018). PROMIS-29 v2.0 profile physical and mental health summary scores. Qual Life Res.

[56] (2021). PROMIS adult profile instruments. PROMIS.

[57] Cella D, Choi SW, Condon DM, Schalet B, Hays RD, Rothrock NE, Yount S, Cook KF, Gershon RC, Amtmann D, DeWalt DA, Pilkonis PA, Stone AA, Weinfurt K, Reeve BB (2019). PROMIS adult health profiles: efficient short-form measures of seven health domains. Value Health.

[58] Lorig KR, Sobel DS, Ritter PL, Laurent D, Hobbs M (2001). Effect of a self-management program on patients with chronic disease. Eff Clin Pract.

[59] Lorig K, Stewart A, Ritter P, González V, Laurent D, Lynch J (1996). Outcome Measures for Health Education and Other Health Care Interventions.

[60] Cyranowski JM, Zill N, Bode R, Butt Z, Kelly MAR, Pilkonis PA, Salsman JM, Cella D (2013). Assessing social support, companionship, and distress: national institute of health (NIH) toolbox adult social relationship scales. Health Psychol.

[61] Swanholm E, McDonald W, Makris U, Noe C, Gatchel R (2014). Estimates of minimally important differences (MIDs) for two patient‐reported outcomes measurement information system (PROMIS) computer‐adaptive tests in chronic pain patients. J Appl Biobehav Res.

[62] (2019). The GLMPOWER procedure. SAS Institute.

[63] Faul F, Erdfelder E, Lang A, Buchner A (2007). G*Power 3: a flexible statistical power analysis program for the social, behavioral, and biomedical sciences. Behav Res Methods.

[64] Bell ML, Fiero M, Horton NJ, Hsu C (2014). Handling missing data in RCTs; a review of the top medical journals. BMC Med Res Methodol.

[65] Wu MC, Carroll RJ (1988). Estimation and comparison of changes in the presence of informative right censoring by modeling the censoring process. Biometrics.

[66] Peugh JL, Toland MD, Harrison H (2023). A tutorial for handling suspected missing not at random data in longitudinal clinical trials. Quant Meth Psych.

[67] Muthén B, Asparouhov T, Hunter AM, Leuchter AF (2011). Growth modeling with nonignorable dropout: alternative analyses of the STAR*D antidepressant trial. Psychol Methods.

[68] Enders CK (2011). Missing not at random models for latent growth curve analyses. Psychol Methods.

[69] Morris T, Kahan BC, White IR (2014). Choosing sensitivity analyses for randomised trials: principles. BMC Med Res Methodol.

[70] Diggle P, Kenward M (1994). Informative drop-out in longitudinal data analysis. J R Stat Soc, C: Appl Stat.

[71] Park S, Kim B (2022). The impact of everyday AI-based smart speaker use on the well-being of older adults living alone. Technol Soc.

[72] Jones VK, Yan C, Shade MY, Boron J, Yan Z, Heselton HJ, Johnson K, Dube V (2024). Reducing loneliness and improving social support among older adults through different modalities of personal voice assistants. Geriatrics (Basel).

[73] Kim D (2023). Can healthcare apps and smart speakers improve the health behavior and depression of older adults? A quasi-experimental study. Front Digit Health.

