# Supplementary material for: Comparison of Smart Display Versus Laptop Platforms for an eHealth Intervention to Improve Functional Health for Older Adults With Multiple Chronic Conditions: Protocol for a Randomized Clinical Trial
Source: JMIR Res Protoc. 2025 Apr 3;14:e64449. doi: 10.2196/64449 (PMC12006769; doi:10.2196/64449)
Supplement: Multimedia Appendix 1 [file resprot_v14i1e64449_app1.docx]

**MULTIMEDIA APPENDIX**

**Figure S1**. ElderTree home screen on the smart display (top) and laptop (bottom) platforms.


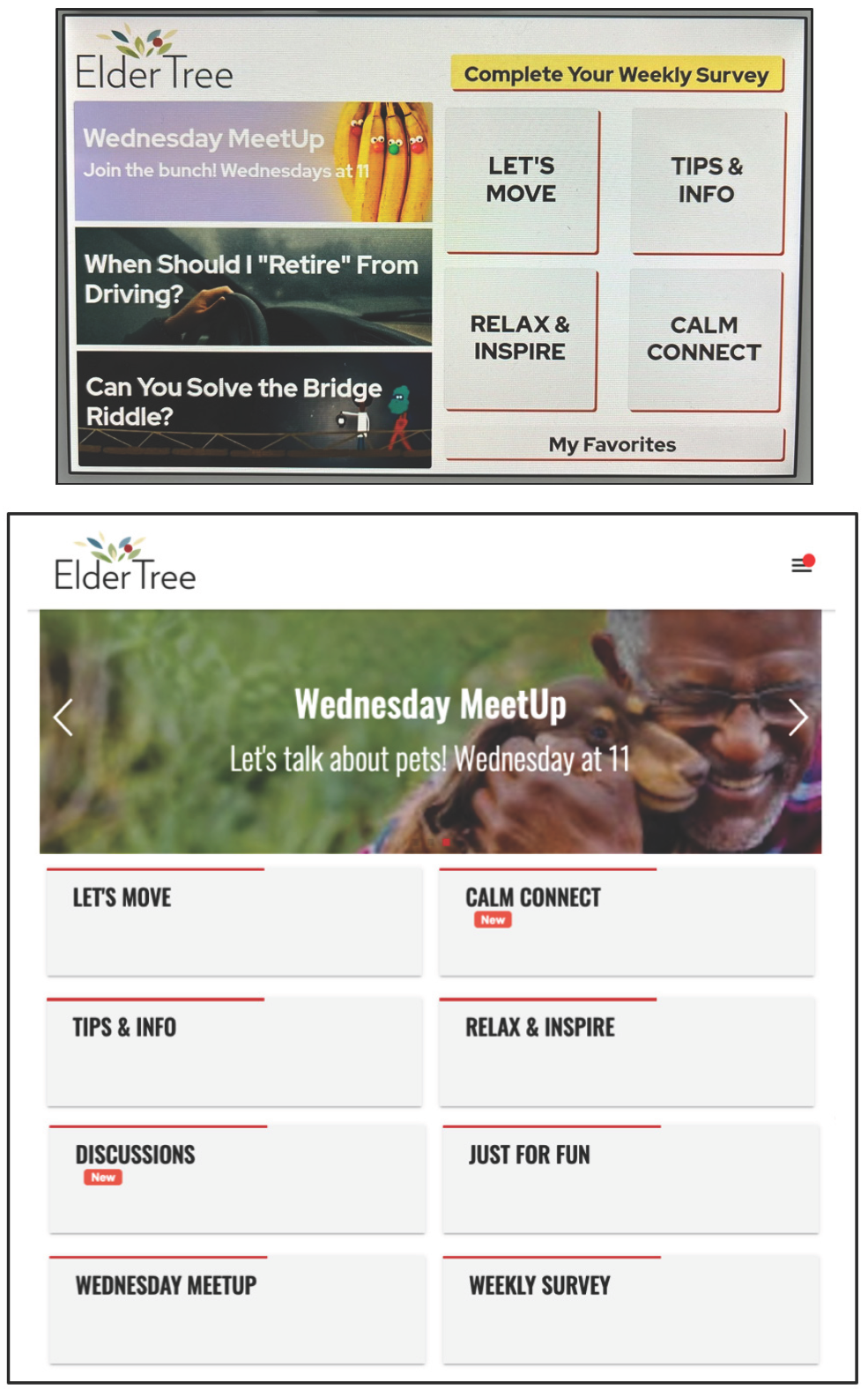

**Figure S2**. Decision tree for determining physical activity group level on ElderTree.

**
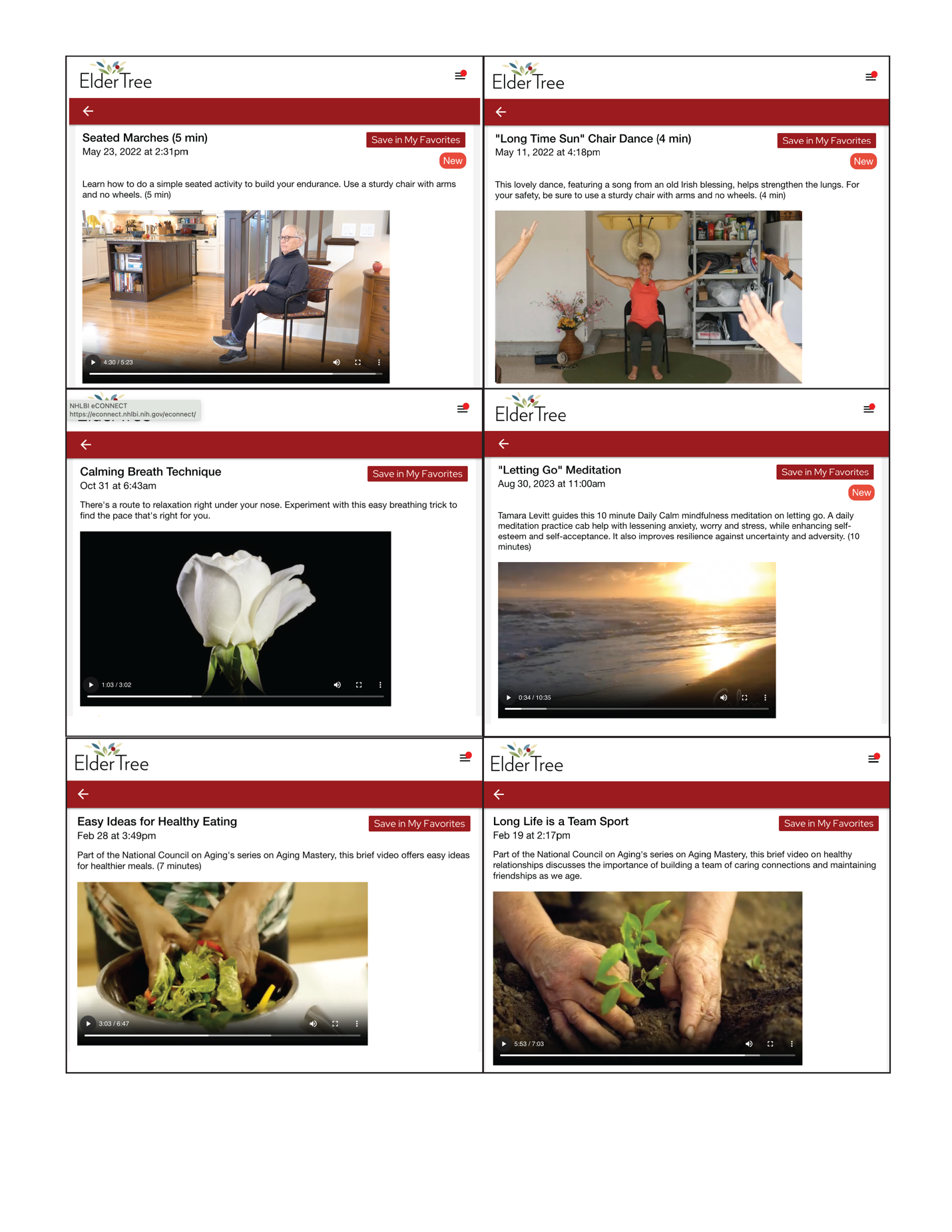
Figure S3.** ElderTree screenshots: Let’s Move (top row), Relax & Inspire (second row), and Tips & Info (bottom row).
