# Supplementary material for: Comparison of Smart Display Versus Laptop Platforms for an eHealth Intervention to Improve Functional Health for Older Adults With Multiple Chronic Conditions: Protocol for a Randomized Clinical Trial
Source: JMIR Res Protoc. 2025 Apr 3;14:e64449. doi: 10.2196/64449 (PMC12006769; doi:10.2196/64449)
Supplement: Multimedia Appendix 2 [file resprot_v14i1e64449_app2.doc]

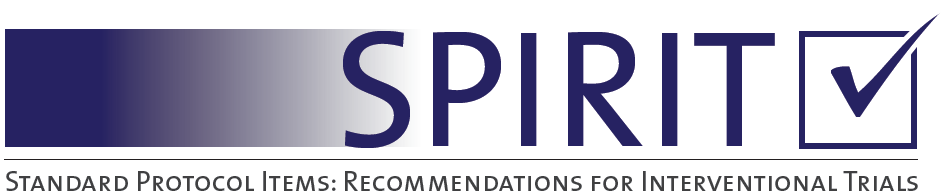


SPIRIT 2013 Checklist: Recommended items to address in a clinical trial protocol and related documents*

| Section/item | Item No | Description | Addressed on page number |
| --- | --- | --- | --- |
| **Administrative information** | | |  |
| Title | 1 | Descriptive title identifying the study design, population, interventions, and, if applicable, trial acronym | ____1_____ |
| Trial registration | 2a | Trial identifier and registry name. If not yet registered, name of intended registry | ____4_____ |
|  | 2b | All items from the World Health Organization Trial Registration Data Set | Addendum at the end of this document_ |
| Protocol version | 3 | Date and version identifier  1.26.2025_RV1 | Here in yellow_____ |
| Funding | 4 | Sources and types of financial, material, and other support | __32-33___ |
| Roles and responsibilities | 5a | Names, affiliations, and roles of protocol contributors | ____1_____ |
|  | 5b | Name and contact information for the trial sponsor  Rebecca Campo, rebecca.campo@nih.gov | Here in yellow |
|  | 5c | Role of study sponsor and funders, if any, in study design; collection, management, analysis, and interpretation of data; writing of the report; and the decision to submit the report for publication, including whether they will have ultimate authority over any of these activities | __32-33___ |
|  | 5d | Composition, roles, and responsibilities of the coordinating centre, steering committee, endpoint adjudication committee, data management team, and other individuals or groups overseeing the trial, if applicable (see Item 21a for data monitoring committee)  David H. Gustafson Sr and Marie-Louise Mares, PIs, direct the research; Klaren Pe-Romashko and John Curtin are responsible for data management and statistical methods; day-to-day management of the study is the responsibility of the Project Director, Gina Landucci. | Here in yellow_____ |
| Introduction |  |  |  |
| Background and rationale | 6a | Description of research question and justification for undertaking the trial, including summary of relevant studies (published and unpublished) examining benefits and harms for each intervention | _5-9______ |
|  | 6b | Explanation for choice of comparators | ____7-9___ |
| Objectives | 7 | Specific objectives or hypotheses | ____8-9___ |
| Trial design | 8 | Description of trial design including type of trial (eg, parallel group, crossover, factorial, single group), allocation ratio, and framework (eg, superiority, equivalence, noninferiority, exploratory) | ____9_____ |
| Methods: Participants, interventions, and outcomes | | |  |
| Study setting | 9 | Description of study settings (eg, community clinic, academic hospital) and list of countries where data will be collected. Reference to where list of study sites can be obtained | ____9_____ |
| Eligibility criteria | 10 | Inclusion and exclusion criteria for participants. If applicable, eligibility criteria for study centres and individuals who will perform the interventions (eg, surgeons, psychotherapists) | ____10____ |
| Interventions | 11a | Interventions for each group with sufficient detail to allow replication, including how and when they will be administered | ___9-10___ |
|  | 11b | Criteria for discontinuing or modifying allocated interventions for a given trial participant (eg, drug dose change in response to harms, participant request, or improving/worsening disease) | ____12____ |
|  | 11c | Strategies to improve adherence to intervention protocols, and any procedures for monitoring adherence (eg, drug tablet return, laboratory tests) | __23-24___ |
|  | 11d | Relevant concomitant care and interventions that are permitted or prohibited during the trial | ___9______ |
| Outcomes | 12 | Primary, secondary, and other outcomes, including the specific measurement variable (eg, systolic blood pressure), analysis metric (eg, change from baseline, final value, time to event), method of aggregation (eg, median, proportion), and time point for each outcome. Explanation of the clinical relevance of chosen efficacy and harm outcomes is strongly recommended | 17-22_____ |
| Participant timeline | 13 | Time schedule of enrolment, interventions (including any run-ins and washouts), assessments, and visits for participants. A schematic diagram is highly recommended (see Figure) | __10-12___ |
| Sample size | 14 | Estimated number of participants needed to achieve study objectives and how it was determined, including clinical and statistical assumptions supporting any sample size calculations | ____21-22_ |
| Recruitment | 15 | Strategies for achieving adequate participant enrolment to reach target sample size | __10-11___ |
| **Methods: Assignment of interventions (for controlled trials)** | | |  |
| Allocation: |  |  |  |
| Sequence generation | 16a | Method of generating the allocation sequence (eg, computer-generated random numbers), and list of any factors for stratification. To reduce predictability of a random sequence, details of any planned restriction (eg, blocking) should be provided in a separate document that is unavailable to those who enrol participants or assign interventions | ___12_____ |
| Allocation concealment mechanism | 16b | Mechanism of implementing the allocation sequence (eg, central telephone; sequentially numbered, opaque, sealed envelopes), describing any steps to conceal the sequence until interventions are assigned | ____12____ |
| Implementation | 16c | Who will generate the allocation sequence, who will enrol participants, and who will assign participants to interventions | ____12____ |
| Blinding (masking) | 17a | Who will be blinded after assignment to interventions (eg, trial participants, care providers, outcome assessors, data analysts), and how | ____12____ |
|  | 17b | If blinded, circumstances under which unblinding is permissible, and procedure for revealing a participant’s allocated intervention during the trial | ____NA___ |
| **Methods: Data collection, management, and analysis** | | |  |
| Data collection methods | 18a | Plans for assessment and collection of outcome, baseline, and other trial data, including any related processes to promote data quality (eg, duplicate measurements, training of assessors) and a description of study instruments (eg, questionnaires, laboratory tests) along with their reliability and validity, if known. Reference to where data collection forms can be found, if not in the protocol | _17-23____ |
|  | 18b | Plans to promote participant retention and complete follow-up, including list of any outcome data to be collected for participants who discontinue or deviate from intervention protocols | _23-24____ |
| Data management | 19 | Plans for data entry, coding, security, and storage, including any related processes to promote data quality (eg, double data entry; range checks for data values). Reference to where details of data management procedures can be found, if not in the protocol | ____24____ |
| Statistical methods | 20a | Statistical methods for analysing primary and secondary outcomes. Reference to where other details of the statistical analysis plan can be found, if not in the protocol | _24-27____ |
|  | 20b | Methods for any additional analyses (eg, subgroup and adjusted analyses) | _26______ |
|  | 20c | Definition of analysis population relating to protocol non-adherence (eg, as randomised analysis), and any statistical methods to handle missing data (eg, multiple imputation) | _24-26____ |
| **Methods: Monitoring** | | |  |
| Data monitoring | 21a | Composition of data monitoring committee (DMC); summary of its role and reporting structure; statement of whether it is independent from the sponsor and competing interests; and reference to where further details about its charter can be found, if not in the protocol. Alternatively, an explanation of why a DMC is not needed Our Center uses the DMC located in the University of Wisconsin’s Institute for Clinical and Translational Research (ICTR). The ICTR DMC will provide services to ensure appropriate measures are in place to promote subject safety, research integrity and compliance with federal regulations and local policies. The DMC members will review protocol-specific reports created by statisticians using data pulled from the Research Electronic Data Capture (REDCap) data management tool. These standard reports will include an overview of study objectives, a review of actual and projected accrual rates, an evaluation of patient demographics for balance of randomization, and a summary of the number and seriousness of adverse events. The board will make recommendations to the Principal Investigator that could include actions of continuation, modification, suspension, or termination. | Here in yellow_____ |
|  | 21b | Description of any interim analyses and stopping guidelines, including who will have access to these interim results and make the final decision to terminate the trial No interim analyses are being conducted. A decision to terminate the trial would be made by PIs David Gustafson Sr and Marie-Louise Mares. | Here in yellow____ |
| Harms | 22 | Plans for collecting, assessing, reporting, and managing solicited and spontaneously reported adverse events and other unintended effects of trial interventions or trial conduct Adverse events are recorded by research staff in REDCap and reported to the DMC and funder as required. | Here in yellow___ |
| Auditing | 23 | Frequency and procedures for auditing trial conduct, if any, and whether the process will be independent from investigators and the sponsor The need for audits is determined independently from investigators and the sponsor by the DMC and UW Clinical Trials Monitoring Service. | Here in yellow__ |
| Ethics and dissemination | | |  |
| Research ethics approval | 24 | Plans for seeking research ethics committee/institutional review board (REC/IRB) approval | ____27___ |
| Protocol amendments | 25 | Plans for communicating important protocol modifications (eg, changes to eligibility criteria, outcomes, analyses) to relevant parties (eg, investigators, REC/IRBs, trial participants, trial registries, journals, regulators) All protocol modifications are reviewed/approved by the University of Wisconsin Minimal Risk Research IRB and communicated our NIH project officer as needed. | Here in yellow__ |
| Consent or assent | 26a | Who will obtain informed consent or assent from potential trial participants or authorised surrogates, and how (see Item 32) | __11-12___ |
|  | 26b | Additional consent provisions for collection and use of participant data and biological specimens in ancillary studies, if applicable | __NA_____ |
| Confidentiality | 27 | How personal information about potential and enrolled participants will be collected, shared, and maintained in order to protect confidentiality before, during, and after the trial | ____24____ |
| Declaration of interests | 28 | Financial and other competing interests for principal investigators for the overall trial and each study site | ___33_____ |
| Access to data | 29 | Statement of who will have access to the final trial dataset, and disclosure of contractual agreements that limit such access for investigators  The PIs (Gustafson Sr and Mares), project director (Landucci), and the statistical core (Pe-Romashko, Curtin) will have access to the final dataset. No contractual agreements limit access for investigators. | Here in yellow_____ |
| Ancillary and post-trial care | 30 | Provisions, if any, for ancillary and post-trial care, and for compensation to those who suffer harm from trial participation None are planned. | Here in yellow_____ |
| Dissemination policy | 31a | Plans for investigators and sponsor to communicate trial results to participants, healthcare professionals, the public, and other relevant groups (eg, via publication, reporting in results databases, or other data sharing arrangements), including any publication restrictions We plan to disseminate results of the trial through publication, without restrictions, regardless of the direction or magnitude of its effects. | Here in yellow_____ |
|  | 31b | Authorship eligibility guidelines and any intended use of professional writers We abide by ICJME authorship criteria: substantial contributions to the conception or design of the work, analysis, or interpretation of data for the work; AND drafting the work or revising it critically for important intellectual content; AND final approval of the version to be published; AND agreement to be accountable for all aspects of the work in ensuring that questions related to the accuracy or integrity of any part of the work are appropriately investigated and resolved. We did not use any professional writers. | Here in yellow_____ |
|  | 31c | Plans, if any, for granting public access to the full protocol, participant-level dataset, and statistical code Once we have completed our analysis, we will make the data available to researchers who contact us and provide evidence that their research has been approved by appropriate review bodies. | Here in yellow_____ |
| Appendices |  |  |  |
| Informed consent materials | 32 | Model consent form and other related documentation given to participants and authorised surrogates  The consent forms are submitted with the manuscript as supplementary files. | Here in yellow_____ |
| Biological specimens | 33 | Plans for collection, laboratory evaluation, and storage of biological specimens for genetic or molecular analysis in the current trial and for future use in ancillary studies, if applicable | Does not apply_____ |

*It is strongly recommended that this checklist be read in conjunction with the SPIRIT 2013 Explanation & Elaboration for important clarification on the items. Amendments to the protocol should be tracked and dated. The SPIRIT checklist is copyrighted by the SPIRIT Group under the Creative Commons “[Attribution-NonCommercial-NoDerivs 3.0 Unported](http://www.creativecommons.org/licenses/by-nc-nd/3.0/)” license.

**Addendum:**

**WHO Checklist**

Primary registry and trial identifying number: ClinicalTrials.govNCT05240534

Date of registration in primary registry: 04 February 2022

Secondary identifying numbers: 4R33HL151870-02

Sources of monetary or material support: National Heart, Lung, and Blood Institute (NHLBI

Primary sponsor: National Heart, Lung, and Blood Institute (NHLBI)

Contact for public queries: DHG Sr, PhD, at [dhgustaf@wisc.edu](mailto:dhgustaf@wisc.edu)

Contact for scientific queries: DHG Sr, PhD, at [dhgustaf@wisc.edu](mailto:dhgustaf@wisc.edu)

Public title: Comparison of Smart Display Versus Laptop Platforms for an eHealth Intervention to Improve Functional Health for Older Adults with Multiple Chronic Conditions: Protocol for a Randomized Clinical Trial

Scientific title: same as above

Countries of recruitment: USA

Health condition(s) or problems(s) studied: challenges from multiple chronic health conditions (such as hypertension, hyperlipidemia, obesity, prediabetes/diabetes, or depression) that threaten the ability of older adults to live independently

Interventions: ElderTree on a Laptop Platform (ET-LT) - Participants in the ET-LT arm receive a touchscreen laptop computer, access to ElderTree (ET), and Internet service for 12 months, and continue with their usual care. ElderTree on a Smart Display Platform (ET-SD) - Participants in the ET-SD arm continue with their usual care and receive a smart display, Internet service, and access to ElderTree for 12 months. The smart display consists of a voice-activated smart speaker and a small visual display that is optionally touch-activated.

Key inclusion and exclusion criteria:

Ages eligible for study: ≥ 60 years

Sexes eligible for study: Both

Accepts eligible volunteers: Yes

Inclusion criteria for older adults: have at least 5 chronic conditions, 3 of which must be hypertension, hyperlipidemia, obesity, prediabetes/diabetes, or depression; live in Madison, Milwaukee, or other area in Wisconsin, USA

Exclusion criteria for older adults: No current psychotic disorder or form of dementia, no acute medical problem requiring immediate hospitalization, no need of an interpreter, and no physical impairments preventing use of a computer or tablet

Study type: Interventional; allocation: randomized; unblinded; two arms, parallel; purpose: improve functional health among older adults

Date of first enrolment: July 2023

Target sample size: 282 (141 per arm) after dropouts

Recruitment status: Recruitment complete

Primary outcome: Functional health

Key secondary outcomes: Physical function, anxiety, depression, social roles, pain interference, health distress, loneliness, unscheduled healthcare use, falls
